# Fast and Ultrasensitive Detection of Monkeypox by a *Pyrococcus furiosus* Argonaute System Coupled with a Short Amplification

**DOI:** 10.3390/v16030382

**Published:** 2024-02-29

**Authors:** Ping He, Wenhao Zhou, Hongping Wei, Junping Yu

**Affiliations:** 1 CAS Key Laboratory of Special Pathogens and Biosafety, Center for Biosafety Mega-Science, Wuhan Institute of Virology, Chinese Academy of Sciences, Wuhan 430207, China; peace192@163.com (P.H.); zyzwh1999@163.com (W.Z.); 2 University of Chinese Academy of Sciences, Beijing 100049, China

**Keywords:** Mpox, *Pf*Ago cleavage, DNA, highly sensitive

## Abstract

Monkeypox virus (MPXV), the pathogen responsible for the infectious disease monkeypox, causes lesions on the skin, lymphadenopathy, and fever. It has posed a global public health threat since May 2022. Highly sensitive and specific detection of MPXV is crucial for preventing the spread of the disease. *Pyrococcus furiosus* Argonaute (*Pf*Ago) is an artificial DNA-guided restriction cleavage enzyme programmable with 5′-phosphorylated ssDNA sequences, which can be developed to specifically detect nucleic acids of pathogens. Here, a *Pf*Ago-based system was established for the detection of MPXV-specific DNA targeting the F3L gene. A short amplicon of 79 bp could be obtained through a fast PCR procedure, which was completed within 45 min. Two 5′-phosphorylation guide DNAs were designed to guide *Pf*Ago to cleave the amplicon to obtain an 18 bp 5′-phosphorylation sequence specific to MPXV, not to other orthopoxviruses (cowpox, variola, and vaccinia viruses). The 18 bp sequence guided *Pf*Ago to cleave a designed probe specific to MPXV to emit fluorescence. With optimized conditions for the *Pf*Ago-MPXV system, it could be completed in 60 min for the detection of the extracted MPXV DNA with the limit of detection (LOD) of 1.1 copies/reaction and did not depend on expensive instruments. Successful application of the *Pf*Ago-MPXV system in sensitively detecting MPXV in simulated throat swabs, skin swabs, sera, and wastewater demonstrated the system’s good performance. The *Pf*Ago platform, with high sensitivity and specificity established here, has the potential to prevent the spread of MPXV.

## 1. Introduction

The Mpox virus (MPXV), a zoonotic virus, first discovered in 1958 and the first human case found in 1970, is highly contagious. Before 2022, MPXV happened rarely and mostly within African countries [1]. Since the outbreak of Mpox in 2022, the infection cases of MPXV have kept growing, posing a public health threat worldwide. There have been a cumulative total of 92,783 confirmed Mpox cases from 1 January 2022 to 30 November 2023, including 171 deaths reported to WHO from 116 countries [2]. In this WHO report published on 22 December 2023, a total of 2024 cases were reported, including one death since September 2022 for the first imported case in China [3]. The MPXV, which can be transmitted through sexual intercourse and direct contacting lesions, saliva, aerosols, as well as contaminated fomite [4], might cause an epidemic. The symptoms of infections caused by MPXV vary from mild to severe, including skin lesions, lymphadenectasis, fever, vomiting, sepsis, etc. [1,4]. The MPXV, a large double-stranded DNA virus (about 197 kb), belongs to the genus of *orthopoxvirus* of the *Poxviridae* family, along with three other viruses: variola virus (VARV), cowpox virus (CPXV), and vaccinia virus (VACV). The genomes of the viruses in the genus *orthopoxvirus* share an identity of at least 96% [5], resulting in cross-protection and the development of a vaccinia vaccine against VARV. The mild symptoms of skin lesions and rashes are close to the infections by other viruses, such as chickenpox virus and herpes viruses, which might interfere with the diagnosis of the infection by the MPXV. Therefore, the specific and rapid diagnosis of the MPXV infection with high sensitivity is important to prevent the spread of the pathogens.

Diagnosis of MPXV infections in the laboratory usually includes two main methods: serological diagnosis and molecular diagnosis. Since there is an immunological cross-reaction between the orthopox viruses, serological diagnosis can be only used to estimate the antibody level against orthopox and infection or vaccination histories and cannot confirm the infection by one of the orthopox viruses. Molecular diagnosis targeting the DNA sequence of the viruses is specific to the target viruses. Regular PCR and real-time PCR methods have been developed to detect the MPXV DNA targeting different genes, such as F3L, G2R, and C3L [6]. Conventional PCR has the merit of not relying on expensive instruments, but its results are not convenient to read. Real-time PCR is fast and convenient to get the final reports but is dependent on complicated equipment as well as skilled personnel.

*Pyrococcus furiosus* Argonaute (*Pf*Ago) belongs to the Argonaute protein family, which is involved in host defense by decomposing intruding nucleic acids [7,8]. In recent years, *Pf*Ago, due to the DNA-guided programmable function through highly efficiently cleaving the base-pairing site at the guide DNA of 10th and 11th bases [7,9,10], has been used for developing *Pf*Ago-mediated Nucleic acid detection platforms to detect some pathogens such as HPV [11], SARS-CoV-2 [12], as well as mutations in the genomes of pathogens [13]. The detections of DNA by *Pf*Ago-mediated systems were usually realized by two steps of cleavage: the first step of cleavage guided by two primers, with the product of this cleavage guiding subsequent cleavage targeting the specific probes. *Pf*Ago can work without amplification steps with low sensitivity, but the sensitivity can be improved by combining it with conventional PCR, RPA, or LAMP [14,15,16,17,18]. Interestingly, because of the programmable function for DNA-guided cleavage, *Pf*Ago was used to connect to an ultrashort PCR to rapidly and highly sensitively detect viruses from clinical samples [19]. It was worth mentioning that the *Tt*Ago protein (*Thermus thermophilus* argonaute, another Agonaute protein) was further utilized to design an artificial nucleic acid circuit to realize amplification-free detection of genes through a cross-cleavage by *Tt*Ago. However, this method exhibited low sensitivity compared to PCR-Ago systems and a relatively long reaction time due to the efficiency of the two Ago units [20]. Therefore, to obtain high sensitivity and rapidity, *Pf*Ago combined with PCR with short amplicons is a good choice to detect viruses.

In this study, a *Pf*Ago platform combined with a short PCR, which was fast and ultrasensitive, was established to detect MPXV. The developed method has the potential to prevent the spread of the Mpox epidemic.

## 2. Materials and Methods

### 2.1. Materials and Reagents

The oligonucleotides were synthesized in Sangon Biotech Co. Ltd. (Shanghai, China). The pET28a plasmid containing the *Pf*Ago gene of 2316 bp [18] and the pUC57 containing the corresponding fragments of VARV, VACV, and CPXV, respectively, were obtained from Gene Create Inc. (Wuhan, China). 2× AceQ Mix for PCR was purchased from Vazyme Biotech Co., Ltd. (Q112, Vazyme, Nanjing, China). *Pf*Ago storage buffer was prepared with 20 mM Tris-HCl (pH 7.5), 300 mM NaCl, and 0.5 mM MnCl_2_. 10× *Pf*Ago cleavage buffer was made by 200 mM HEPES (pH 7.5), 2.5 M NaCl, and 5 mM MnCl_2_, which was divided into aliquots of 1 mL each. All chemicals were of analytical grade and purchased from local commercial suppliers unless otherwise specified. RNase-Free water was acquired from Yuanye Biotech. (Shanghai, China). The deionized water (18.2 MΩ) was used to prepare all buffers.

### 2.2. Expression and Purification of PfAgo

The pET28a plasmid containing the *Pf*Ago gene with a his-tag at the N-terminal was transformed into the *E. coli* BL21(DE3) pLysS strain to express the protein. Isopropyl β-D-1-thiogalactopyranoside (IPTG) at a final concentration of 1 mM was added to induce the expression of *Pf*Ago by incubating at 16 °C overnight. The 6× His-tagged *Pf*Ago was purified by Ni sepharose (GE Healthcare, Marlborough, MA, USA) with a gradient imidazole elution method. Subsequently, the purified protein was dialyzed in *Pf*Ago storage buffer and concentrated by a 50 kDa ultrafiltration tube (Merck, MA, USA) centrifuged at 4000× *g*. SDS-PAGE was used to confirm the expression and the purification of *Pf*Ago, while the concentration of the protein was determined using A280 measurements on a Nanodrop spectrophotometer.

### 2.3. Confirmation of the Cleavage Activity of the Recombinant PfAgo

Two single-stranded DNA (ssDNA) sequences and 10 guide DNAs (listed in Table S1) covering the full amplicon region of 79 bp on the F3L gene of MPXV were designed to confirm the cleaving activity of *Pf*Ago/gDNA complexes to corresponding ssDNAs. Additionally, gDNA2 was shifted 6 bases to the right compared to gDNA1, and so on. And there was a 10-base overlap between the adjacent gDNAs. The *Pf*Ago cleavage system included 20 pmol recombinant *Pf*Ago, 10 pmol ssDNA, 10 pmol guide DNA, and *Pf*Ago reaction buffer with a final volume of 20 μL. After reaction at 95 °C for 30 min, the products were subjected to 20% Urea-PAGE for electrophoresis in 1× TBE. SYBR gold was used to dye the gel for imaging on an imaging system (ChemiDoc XRS+, BioRad, Hercules, CA, USA).

### 2.4. PCR of the 79 bp Amplicon

The primers named MPXV-F3L-F and MPXV-F3L-R (listed in Table S1) for the MPXV-specific fragment of 79 bp were referred to in a previous study [21]. The MPXV from cell culture supernatant of 200 μL was inactivated at 60 °C for 35 min on a metal bath (MK2000-2E, Allsheng Inc, Hangzhou, China), and then the DNA of the inactivated MPXV was extracted by a virus DNA/RNA extraction kit on a magnetic beads-based DNA/RNA Extractor NPA-32p (Bioer Tech., Hangzhou, China), to serve as the template for PCR. The PCR system of 20 µL included 10 µL of the 2× AceQ Mix (Q112, Vazyme, Nanjing, China), 0.6 µL 10 μM of the primers, 3.8 μL of RNase-free water, and 5 µL of the template. The PCR procedure involved initial pre-denaturation at 95 °C for 1 to 5 min for optimization, followed by 40 cycles of 95 °C for 10 s and 58 °C for 10–30 s for optimization. The PCR product was subjected to non-denaturing PAGE to check the target bands.

### 2.5. The Procedure of the PfAgo-MPXV System

The procedure of the *Pf*Ago-MPXV system comprised two steps: PCR and cleavage. The PCR mixture was prepared and processed as described above. After PCR with the optimized conditions (pre-denaturation at 95 °C for 1 min followed by 40 cycles of 95 °C for 10 s and 58 °C for 10 s, completed within 45 min), guide DNAs-guided cleavage of target DNA by *Pf*Ago was performed. The *Pf*Ago cleavage system of 20 μL containing 2 μL of 10× *Pf*Ago reaction buffer, MnCl_2_ ranging from 0.25 mM to 8 mM for optimization, gDNA3 and gDNA6 at concentrations ranging 0.16 μM to 5 μM each for optimization, the reporter probe double-labeled 3′-BHQ1 and 5′-FAM-6 ranging from 0.125 μM to 2 μM for optimization and *Pf*Ago ranging from 0.31 μM to 10 μM for optimization, was added into 20 μL PCR product. During the process of the *Pf*Ago cleavage reaction at 95 °C, the fluorescence of the system was recorded every minute for 30 min on the Bio-Rad CFX96 instrument (Bio-Rad, Hercules, CA, USA).

### 2.6. Determination of the Copy Number of Extracted MPXV DNA by ddPCR

ddPCR was used to determine the copy number of the inactivated MPXV. Droplets were generated by an automated droplet generator, and the signal reading was completed on QX200 AutoDG, BioRad. ddPCR Supermix for Probes (No dUTP) was used. The ddPCR system consisted of 10 μL 2× Supermix for probes and 250 nM primer and probe concentrations, respectively, 5 μL extracted DNA from the diluted inactivated MPXV and DNase-free water to a final volume of 20 μL. The primers/probe (listed in Table S1) used in ddPCR were the same as the primers (F3L-F and F3L-R) and the FAM-labeled reporter/probe (F3L-P) used in the *Pf*Ago-MPXV detection system.

### 2.7. The Detection of MPXV in Simulated Samples

Serum samples, throat swabs, and skin swabs collected from healthy volunteers, as well as wastewater samples from a local hospital wastewater system (without positive signals), were mixed with inactivated MPXV through the volume ratio of 100:1. The inactivated MPXV with the final concentrations of 400,000, 40,000, 4000, 400 copies/mL added in the four types of samples were prepared. Then, 400 μL of the simulated samples were extracted, and the purified DNAs were subjected to MPXV detection by the *Pf*Ago-MPXV system with 1.25 μM of *Pf*Ago, 0.31 μM of each guide DNA, 2 mM Mn^2+^ and 0.5 μM reporter. The fluorescence of the system was read at 15 min after cleavage by *Pf*Ago.

### 2.8. Statistics and Reproducibility

Probit analysis has been adopted for determining qPCR LODs, which is robust and empirical and commonly used in the detection of pathogens [22]. Probit analysis was used to determine the 95% LOD of the *Pf*Ago-MPXV system, which was performed on MedCalc software 22.021 (MedCalc Software Ltd., Ostend, Belgium).

## 3. Results

### 3.1. Design of the PfAgo-MPXV System for the Detection of MPXV

**Figure 1:**
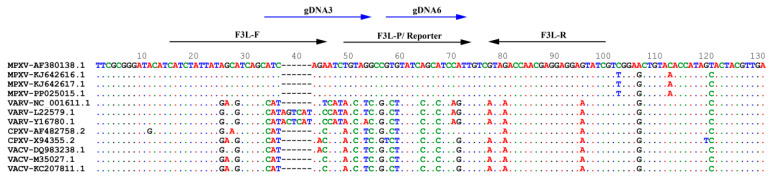
Comparison of the F3L gene of MPXV (Monkeypox virus strain Zaire-96-I-16: AF380138.1; Monkeypox virus strain PCH: KJ642616.1; Monkeypox virus strain Nigeria-SE-1971: KJ642617.1; Monkeypox virus isolate MPXV/PT0772/2023: PP025015.1) with the corresponding regions of VARV (strain India-1967: NC_001611.1; strain Bangladesh-1975: L22579.1 and Y16780.1; Garcia-1966: Y16780.1), CPXV (Brighton Red: AF482758.1; Cowpox virus strain GRI-90: X94355.2), VACV (Vaccinia virus strain MVA-BN: DQ983238.1; Vaccinia virus Copenhagen: M35027.1; Vaccinia virus strain TianTan clone TP5: KC207811.1) and the sequences of the primers and guide DNAs were localized.

MPXV F3L, which was conserved for all MPXV clades [6,23] and had been used to differentiate MPXV from other orthopox viruses [21], was selected to design the guide DNAs for the detection system. As shown in Figure 1, the F3L region (position of F3L gene from 301 to 418) of four MPXV genomes of MPXV_AF380138.1 (cladeI), KJ642616.1 (cladeIIa), KJ642617.1 (cladeIIb early) and PP025015.1 (cladeIIb-2023) were compared with other orthopox viruses: VARV, CPXV and VACV. The displayed sequences of F3L regions of the four MPXV strains were exactly the same and different from the corresponding regions of VARV, VACV, and CPXV. To realize the specific detection of MPXV in the *Pf*Ago-MPXV system, the amplification region was located in a position where there are many variations compared to other orthopox viruses but no mutations among different strains of MPXV. In order to shorten the PCR time, it is preferable to select a shorter amplification region while ensuring amplification specificity and efficiency as much as possible. Therefore, primers of a 79 bp amplicon were chosen [21]. The localization of the primers is exhibited in Figure 1.

**Figure 2:**
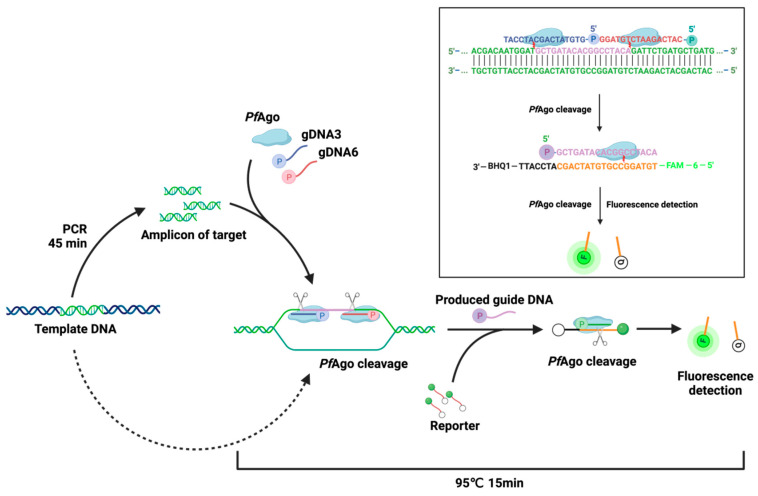
The principle of the *Pf*Ago-MPXV platform is to detect MPXV. The scheme was produced on www.biorender.com, accessed on 6 May 2023.

The work scheme of the *Pf*Ago-MPXV platform is revealed in Figure 2. The nucleotide with the MPXV genome as a template was used to amplify a 79-bp amplicon. Two 5′-phosphorylated guide DNAs guided *Pf*Ago to cleave the amplicon to obtain a 5′-phosphorylated 18-bp sequence as a produced guide DNA, which in turn guided *Pf*Ago to cut the FAM-labeled reporter at the binding position between 10th and 11th bases of the produced guide DNA. The fluorescence signal released by the cleaved reporter was recorded by a real-time fluorescence PCR system.

### 3.2. Evaluation of the PfAgo-MPXV Platform

**Figure 3:**
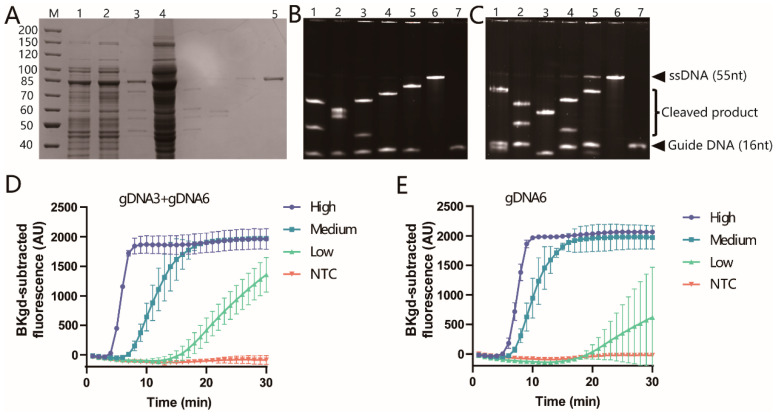
The expression of *Pf*Ago protein and evaluation of the activity of *Pf*Ago. (**A**) SDS-PAGE of expressed *Pf*Ago protein. The unit of the numbers was kDa. Lane M: Marker; Lane 1: the bacterial lysate before induction; Lane 2: the bacterial lysate after induction; Lane 3: the supernatant of the bacterial lysate heated at 80 °C; Lane 4: the precipitate of the bacterial lysate heated at 80 °C; lane 5: the purified *Pf*Ago. (**B**) Urea-PAGE of the cleavage of ssDNA template 1 guided by gDNA1 to gDNA5 from Lane 1 to Lane 5, respectively. Lane 6: ssDNA template 1; Lane 7: gDNA5. (**C**) Urea-PAGE of the cleavage of ssDNA template 2 guided by gDNA6 to gDNA10 from Lane 1 to Lane 5, respectively. Lane 6: ssDNA template 2; Lane 7: gDNA10. The two arrowheads indicated the un-cleaved ssDNA and the guide DNA, respectively. The brace indicated the cleaved products on the bands. (**D**) gDNA3 and gDNA6 were used to guide the cleavage by *Pf*Ago. (**E**) The gDNA6 was individually utilized to guide the cleavage by *Pf*Ago, and the probe worked well for different concentrations of MPXV DNA. Error bars indicate the standard deviation of three replicates.

Firstly, the expression of *Pf*Ago protein was confirmed by SDS-PAGE, as displayed in Figure 3A, which indicated that the correct size (~90 kDa) of the protein was expressed. To verify the activity of *Pf*Ago, 10 5′-phosphorylated ssDNA sequences as guide DNA and two ssDNA sequences as cleaved templates (The sequences used in this study were listed in Table S1 in the supplementary materials) were synthesized based on the sequence of the F3L gene. Urea-PAGE was used to evaluate the cleavage activities of *Pf*Ago on the two ssDNA templates guided by the ten guide DNAs, the results of which were shown in Figure 3B,C. All the ten guide DNAs guided the expressed *Pf*Ago to efficiently cleave the templates, demonstrating good activities of the *Pf*Ago protein.

According to the high cleavage activity of the complexes and the region of the multiple base mutations as well as the synthesized reporter sequence, two guide DNA sequences (gDNA3 and gDNA6) were chosen, and the locations of the sequences were indicated on the F3L gene in Figure 1. To ensure whether the system could work, an initial test was performed by applying gDNA3 and gDNA6 together, as well as individually, combined with the probe for detecting MPXV DNA. As revealed in Figure 3D,E, gDNA3, and gDNA6 together or gDNA6 separately with the probe could guide *Pf*Ago effectively cleaving, and different concentrations of MPXV DNA gave obvious fluorescence signals, and the negative controls showed no signal. Single gDNA3 did not work, and no fluorescence signals were obtained. Figure 3D,E demonstrated that gDNA3 and gDNA6 together could achieve a better and earlier fluorescence signal than gDNA6 separately. Therefore, two gDNAs were adopted for the *Pf*Ago-MPXV system.

### 3.3. Optimization of the PfAgo-MPXV System

**Figure 4:**
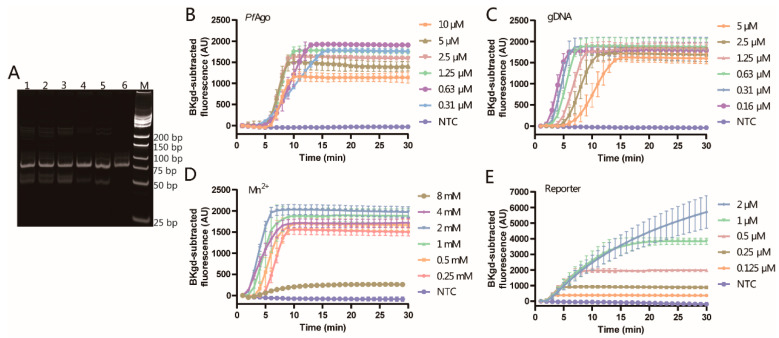
Optimization of the *Pf*Ago-MPXV system. (**A**) Optimization of the annealing/extension time and pre-denaturation time. Lanes 1 to 4 corresponded to the pre-denaturation time of 0 min, 1 min, 3 min, and 5 min with an annealing/extension time of 30 s, and Lanes 4 to 6 corresponded to the annealing/extension time of 30 s, 20 s, and 10 s following a pre-denaturation of 5 min. Lane M is the marker. (**B**) Optimization of the final *Pf*Ago protein concentration. (**C**) Optimization of the final gDNAs concentration. (**D**) Optimization of the final Mn^2+^ concentration. (**E**) Optimization of the final reporter concentration. Error bars indicate the standard deviation of three replicates.

Firstly, to minimize amplification time, two steps of amplification, where annealing and extension were finished at one temperature, were adopted. According to the manufacturer’s instruction of the mixture for amplification, pre-denaturation of 5 min, denaturation of 10 s, and annealing/extension of 30 s were recommended. Due to the shortness of the amplicon, the pre-denaturation time and the annealing/extension time were optimized to obtain the 79 bp amplicon since the denaturation time was very short. As displayed in Figure 4A, at the annealing/extension time of 10 s, the band obtained was clear and bright, which was similar to the band at the annealing/extension time of 30 s. The band with no pre-denaturation step showed a similar brightness to the band with a pre-denaturation of 5 min. Considering the complexity of some types of samples, the pre-denaturation of 1 min was chosen for PCR. Therefore, the annealing/extension time of 10 s and the pre-denaturation time of 1 min was adopted for amplification of the 79 bp amplicon of the target.

Furthermore, to obtain optimal performance for the detection of MPXV DNA, the concentrations of *Pf*Ago, guide DNAs, Mn^2+^, and reporter/probe were optimized to achieve high and stable fluorescence within a time as short as possible.

*Pf*Ago catalyzed cleavage of the target DNA guided by guide DNAs, so the amount of *Pf*Ago is pivotal for the detection system. The concentrations of *Pf*Ago for optimization ranged from 0.31 to 10 μM with the two guide DNAs of 2.5 μM each, Mn^2+^ of 0.25 mM, and the reporter of 0.5 μM to detect the amplicon from the template of 80 copies. As shown in Figure 4B, all the tested concentrations of *Pf*Ago can effectively detect 80 copies/reaction, and as the *Pf*Ago concentration increases, the time to reach the plateau is reduced. The fluorescence can reach plateaus in the shortest time with similar fluorescence values at the concentrations of 1.25, 2.5, and 5 μM. Therefore, *Pf*Ago of 1.25 μM was adopted for further experiments.

Then, guide DNAs, guiding the cleavage of *Pf*Ago to the target DNA, played a crucial role in the detection system. The concentrations of the two guide DNAs were kept the same and optimized from 0.16 μM to 5 μM with *Pf*Ago of 1.25 μM, Mn^2+^ of 0.25 mM and the reporter probe of 0.5 μM. The time to the plateau of the fluorescence was reduced with the decline of the concentrations of the guide DNAs, as indicated in Figure 4C. The shrink of the time to the plateau was observed, and the time at 0.16 μM of guide DNAs was close to the time at 0.31 μM of guide DNAs. After completing the guide mission, the guide DNAs would be detached and become available for the next guide. For the detection system, the guide DNAs will not decrease during the cleavage, while initial guide DNAs should be enough to initialize the first round of cleavage. Therefore, 0.31 μM was picked for the optimization of other conditions.

Metal ions function as a cofactor to many enzymes related to DNA cleavage or DNA amplification. Mn^2+^ is needed to help *Pf*Ago mediate the cleavage of the target DNA guided by guide DNA [7]. Here, the concentration of Mn^2+^ was tested, varying from 0.25 mM to 8 mM, to determine the appropriate concentration of Mn^2+^ for the *Pf*Ago-MPXV detection system, which utilized *Pf*Ago of 1.25 μM, guide DNAs of 0.31 μM and the reporter probe of 0.5 μM. As exhibited in Figure 4D, the time to the plateau was initially decreasing and then increasing with the increment of the concentration of Mn^2+^. Similarly, the fluorescence intensity first increased and then decreased with the increasing concentration of Mn^2+^. At Mn^2+^ of 2 mM, the shortest time to the plateau and the highest fluorescence indicated that Mn^2+^ of 2 mM was a preferred concentration for further experiments.

Here, the reporter is a sequence of DNA doubly labeled with 3′-BHQ1 and 5′-FAM-6, providing the signal of the MPXV detection system after the reporter cleaved. The concentration of the reporter was optimized from 0.125 μM to 2 μM with *Pf*Ago of 1.25 μM, guide DNAs of 0.31 μM and Mn^2+^ of 2 mM. The enhanced concentration of the reporter produced higher fluorescence but also prolonged the time to reach the plateau or even prevented the plateau from forming altogether, as shown in Figure 4E. A concentration of 0.5 μM, which has an obviously fast plateau and enough fluorescence, was adopted for the detection system.

### 3.4. Sensitivity and Specificity of the PfAgo-MPXV System

**Figure 5:**
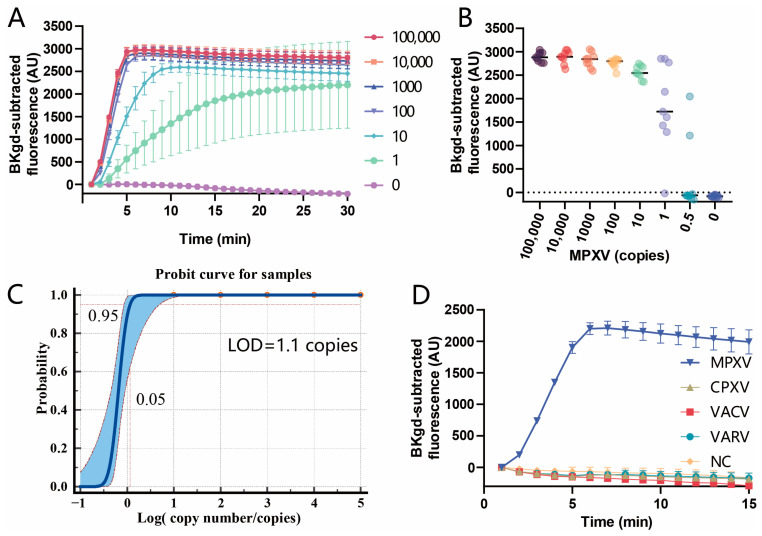
Assessment of sensitivity and specificity of the *Pf*Ago-MPXV system. (**A**) The tracing curves of different copies per reaction were monitored for 30 min. (**B**) Recording of the fluorescence values for each dilution at 15 min with nine replicates. (**C**) The probit curve of the probability of each dilution with nine repeats vs. the corresponding copies was depicted and fitted by a sigmoidal model. (**D**) The system was used to test the plasmids containing the fragments corresponding to VARV, VACV, and CPXV, respectively. The error bars showed the standard deviations from three repeats.

By using the conditions obtained above *Pf*Ago of 1.25 μM, guide DNAs of 0.31 μM, Mn^2+^ of 2 mM, and the reporter of 0.5 μM, the sensitivity was determined by serial dilution of MPXV DNA extracted from the inactivated MPXV, ranging from 0.5 copies to 100,000 copies per reaction. The copy number of the extracted DNA was determined by ddPCR (the data of ddPCR was displayed in Figure S1 in the Supplementary Materials). Firstly, the tracing curves for all the diluted DNA solutions were recorded with an interval of 1 min. As shown in Figure 5A, for the copies per reaction of more than 10, the plateaus of the curves were present within 10 min, but for 1 copy/reaction, fluorescence was much lower at 10 min than at 15 min. Therefore, in order to achieve high sensitivity, the *Pf*Ago cleaving time of 15 min was adopted. Then, 9 repeats for each dilution were performed to test the sensitivity of the *Pf*Ago-MPXV system. As displayed in Figure 5B,C, the probit sigmoid curve fitting for the probability of the six serial dilutions provided the 95% LOD of 1.1 copies per reaction, which was better than the limit of current PCR-based methods for MPXV (3.5 copies/reaction) [6]. Meanwhile, the specificity of the detection system was examined by detecting the plasmids of 1 × 10^7^ copies/μL, each containing the fragments corresponding to VARV, VACV, and CPXV, respectively. The results in Figure 5D indicated that the detection system was specific to MPXV and not interfered with by the other three orthopox viruses.

### 3.5. Detection of MPXV in Simulated Samples

**Figure 6:**
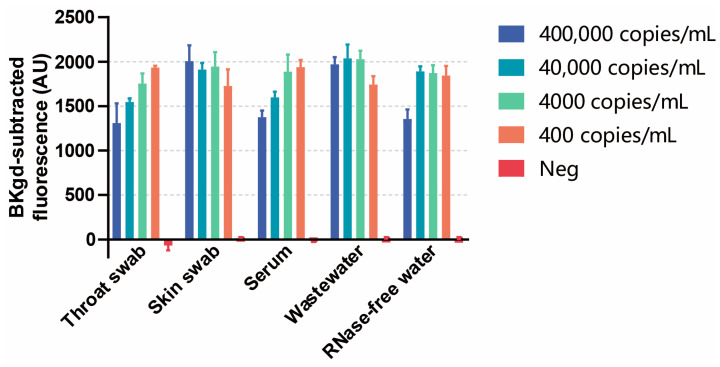
Detection of four different concentrations of MPXV in simulated throat swabs, skin swabs, serum samples, and wastewater samples (from 400 to 400,000 copies/mL). The standard deviations of the means were shown as error bars from triplicates.

By adding four different concentrations of inactivated MPXV to throat swabs, skin swabs, serum samples, and wastewater, a series of simulated samples were tested by the *Pf*Ago-MPXV system. As illustrated in Figure 6, for all the simulated positive samples, the fluorescence values are obviously much higher than the negatives, indicating the effectiveness of the *Pf*Ago-MPXV system in detecting MPXV DNA in the four different types of samples.

## 4. Discussions

For rapid, sensitive, and convenient detection of MPXV infections, a *Pf*Ago-based DNA detection method was developed. The length of the amplicon of 79 bp was perfect, covering the regions of a 25 bp forward primer and a 23 bp reverse primer. The remaining just contained the length of the two guide DNAs of 16 bp each, with a small overlap with the primers. Therefore, the 79 bp amplicon adopted here brought the rapidity of the PCR step before the step of *Pf*Ago cleaving, which was completed within 45 min. Besides, due to the high efficiency of the designed guide DNAs and a resultant guide DNA, the *Pf*Ago system was accomplished in 15 min with a high sensitivity of LOD 1.1 copies/reaction, which was better than the limit of current PCR-based methods for MPXV (3.5 copies/reaction). The *Pf*Ago-MPXV system is specific to MPXV because of the specificity of the guide DNAs to the most various fragments of the F3L gene of MPXV, different from the other orthopox viruses, including VARV, VACV, and CPXV. Only a regular PCR machine and a fluorescent spectrometer are needed for the *Pf*Ago-MPXV system, which is not dependent on expensive instruments. Additionally, the *Pf*Ago system can be realized without PCR or with small cycles of PCR for highly positive samples [19].

As for the optimization of several conditions, the orthogonal test is a good method to get really optimized conditions, but design tables can only include a limited number of factors and level combinations, leading to incomplete results. A full factorial experiment contains a lot of work and requires substantial resources. Instead, a single-factor experiment can be used to get optimized conditions, which was adopted in many publications [14,24,25]. Besides, for one factor, the trend of the results caused by varying another factor is consistent according to a previous study [18]. Therefore, relatively optimized conditions can be obtained through a single-factor experiment, and through this method, a good sensitivity was achieved for the developed *Pf*Ago system. Furthermore, from the curves for the optimization of *Pf*Ago shown in Figure 4B, the cleavages were faster and more effective at low concentrations of *Pf*Ago than those at high concentrations, indicating that the concentration of *Pf*Ago was not consumed and kept constant during the cleavage reactions and excessive *Pf*Ago might inhibit the cleavage. In addition, gDNA was also not consumed during the process. After finishing the first round of guides to cleave, gDNA started to guide the next round of guide and cleavage. Too much gDNA3 and gDNA6 might interfere with the binding of the gDNAs to the target amplicon, as displayed in Figure 4C, where a lower concentration of gDNAs resulted in a shorter time to the plateau. Similarly, the small amount of gDNA produced from the first cleavage by *Pf*Ago guided by gDNA3 and gDNA6 was enough to guide the *Pf*Ago to cleave the reporter due to no consumption during the cleavage.

Meanwhile, the system could be further improved. Firstly, the fluorescence varied a lot for different types of samples, and the fluorescence intensity did not exhibit a linear relationship to the concentration of the DNA template. The same situation happened for other Ago enzyme-based detection systems [12,13]. Therefore, the *Pf*Ago-MPXV detection system, like most diagnostic methods, is a qualitative rather than quantitative method. Furthermore, preamplification by PCR is crucial for detecting low viral loads in samples, and a high concentration of Mn^2+^ was necessary for the cleavage activity of the *Pf*Ago protein, while Mn^2+^ can interfere with the PCR reaction. As a result, the two-step reaction of the *Pf*Ago-MPXV system cannot be realized in a single PCR tube, which limits its field application, thus highlighting the need for designing specialized PCR tubes in the future. Additionally, for specificity tests, plasmids containing corresponding fragments instead of whole viruses were conducted because of the absence of other orthopox viruses in our institute. Moreover, real clinical samples of different types should be tested for clinical application of the *Pf*Ago-MPXV system.

## 5. Conclusions

A *Pf*Ago system combined with a short PCR was designed to specifically detect MPXV. A perfect length of 79 bp of the amplicon and high effectiveness of the cleavage of the target sequences of *Pf*Ago by the guide DNAs brought out the rapidity of the detection system within 60 min, where 45 min was for PCR and 15 min for the *Pf*Ago-based cleavage. The developed *Pf*Ago-MPXV system has a very high sensitivity with LOD 1.1 copies/reaction, which is almost the theoretical limit of the PCR-based methods. The results of detecting MPXV for different types of simulated samples testified to the good performance of the system. The built method can be potentially developed into an MPXV detection kit clinically.

## Data Availability

The raw data supporting the conclusions of this article will be made available by the authors on request.

## Supplementary Materials

The following supporting information can be downloaded at: https://www.mdpi.com/article/10.3390/v16030382/s1, Figure S1: The copy number of the extracted DNA was decided by ddPCR. (A) Scattering dots of the ddPCR results. (B) The copy concentration in the 20 μL ddPCR reaction mixture. D12, E12, and F12 were triplicate. G12 was the negative control. The DNA was extracted from the sample 100-fold diluted from inactivated MPXV. By analyzing the results of ddPCR, the concentration of viral gene copies is determined to be 400,000 copies/mL; Table S1: The sequences used in this study.
