# Supplementary material for: Fast and Ultrasensitive Detection of Monkeypox by a Pyrococcus furiosus Argonaute System Coupled with a Short Amplification
Source: Viruses. 2024 Feb 29;16(3):382. doi: 10.3390/v16030382 (PMC10975468; doi:10.3390/v16030382)
Supplement: Supplementary file 1 [file viruses-16-00382-s001.zip › viruses-2855610-supplementary.pdf]

Supplementary Materials

Table S1. The sequences used in this study

Name	Sequence (5'-3')
gDNA1	TTATAGCATCAGCATC
gDNA2	CATCAGCATCAGAATC
gDNA3	CATCAGAATCTGTAGG
gDNA4	AATCTGTAGGCCGTGT
gDNA5	TAGGCCGTGTATCAGC
gDNA6	GTGTATCAGCATCCAT
gDNA7	CAGCATCCATTGTCGT
gDNA8	CCATTGTCGTAGACCA
gDNA9	TCGTAGACCAACGAGG
gDNA10	ACCAACGAGGAGGAGT
F3L-forward	CATCTATTATAGCATCAGCATCAGA
F3L-reverse	GATACTCCTCCTCGTTGGTCTAC
F3L-reporter/probe	TGTAGGCCGTGTATCAGCATCCATT
79 bp amplicon	CATCTATTATAGCATCAGCATCAGAATCTGTAGGCCGTGTATCAGCA TCCATTGTCGTAGACCAACGAGGAGGAGTATC
ssDNA1	GATGCTGATACACGGCCTACAGATTCTGATGCTGATGCTATAATAG ATGATGTAT
ssDNA2	CCGACGATACTCCTCCTCGTTGGTCTACGACAATGGATGCTGATAC ACGGCCT

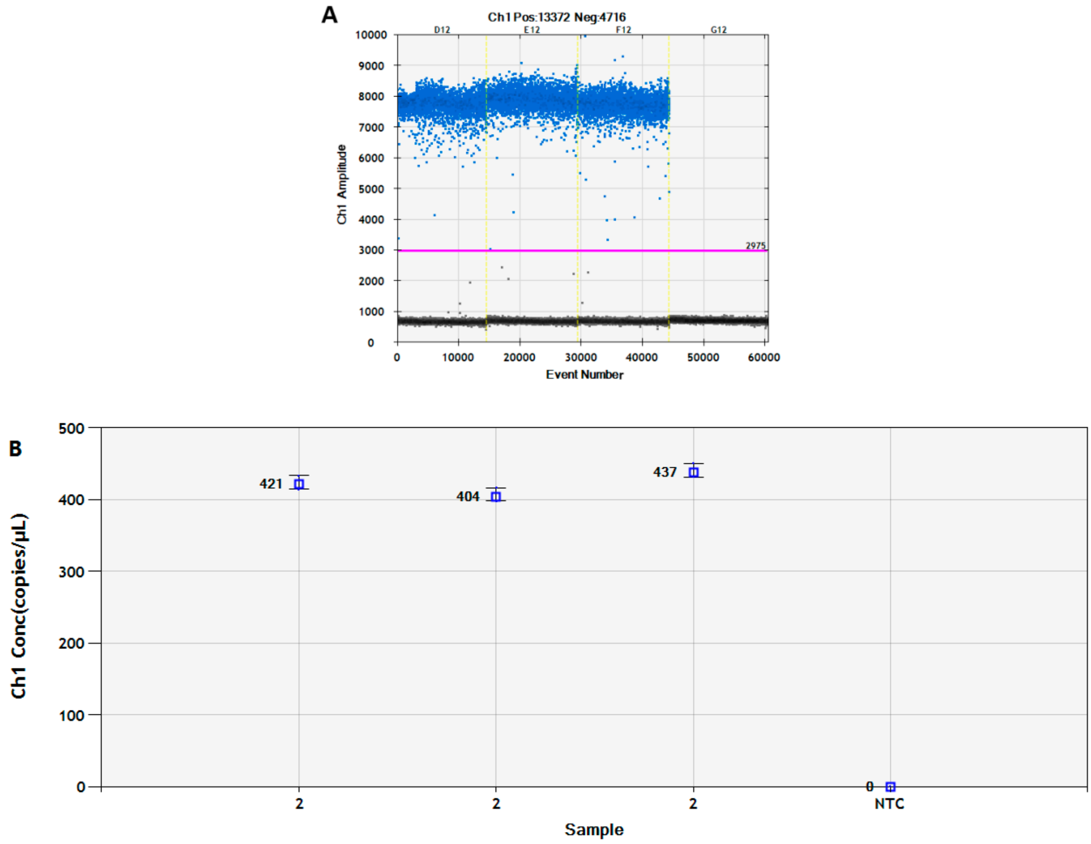

Figure S1. The copy number of the extracted DNA was decided by ddPCR. (A) Scattering dots of the ddPCR results. (B) The copy concentration in the 20 μL ddPCR reaction mixture . D12, E12, and F12 were triplicate. G12 was the negative control. The DNA was extracted from the sample 100-fold diluted from inactivated MPXV. By analyzing the results of ddPCR, the concentration of viral gene copies is determined to be 400,000 copies/mL.
